# Muscle Fatigue and Swimming Efficiency in Behind and Lateral Drafting

**DOI:** 10.3389/fphys.2022.835766

**Published:** 2022-03-03

**Authors:** Luca Puce, Karim Chamari, Lucio Marinelli, Laura Mori, Marco Bove, Emanuela Faelli, Marco Fassone, Filippo Cotellessa, Nicola Luigi Bragazzi, Carlo Trompetto

**Affiliations:** ^1^Department of Neuroscience, Rehabilitation, Ophthalmology, Genetics, Maternal and Child Health, University of Genoa, Genoa, Italy; ^2^Aspetar, Orthopaedic and Sports Medicine Hospital, FIFA Medical Centre of Excellence, Doha, Qatar; ^3^ISSEP Ksar-Said, La Manouba University, Manouba, Tunisia; ^4^IRCCS Ospedale Policlinico San Martino, Genoa, Italy; ^5^Department of Experimental Medicine, Section of Human Physiology, University of Genoa, Genoa, Italy; ^6^Centro Polifunzionale di Scienze Motorie, University of Genoa, Genoa, Italy; ^7^Laboratory for Industrial and Applied Mathematics (LIAM), Department of Mathematics and Statistics, York University, Toronto, ON, Canada

**Keywords:** open water, triathlon, surface electromyography, motor units, hydrodynamic interactions, training

## Abstract

Drafting in swimming is a tactic in which an athlete (drafter) swims in the wave of another athlete (leader). Our aim was to compare the effects of this tactic on the drafter, as far as muscle fatigue, muscle activity, and swimming efficiency are concerned. Fifteen drafters performed three 200 m front crawl trials at a controlled submaximal pace in three configurations: Behind Drafting (BD), Lateral Drafting (LD), and Free Swimming (FS). Muscle fatigue, muscle activity, and swimming efficiency were obtained by surface electromyography (EMG) and video analysis from flexor carpi radialis, triceps brachii, latissimus dorsi, and rectus femoris muscles. The outcome measures were: time slope of Mean Frequency (MNF), for muscle fatigue; time slope of Root Mean Square (RMS), for muscle activity; and Stroke Index (SI) for swimming efficiency. Negative variations of MNF were 5.1 ± 1.7%, 6.6 ± 4.1%, and 11.1 ± 2.7% in BD, LD, and FS, respectively. Statistical significance was found for all cases except for the rectus femoris. Positive variations of RMS were 3.4 ± 1.2%, 4.7 ± 2.7%, and 7.8 ± 4.6% in BD, LD, and FS, respectively. Statistical significance was found only for the slopes of latissimus dorsi in FS and LD. The largest mean in SI was measured in the BD (2.01 m^2^/s), while the smallest was measured in the FS (1.86 m^2^/s). BD was found to be the best swimming configuration, in terms of lower muscle fatigue and higher swimming efficiency. Also, LD resulted to be advantageous with respect to FS.

## Highlights

- In both Behind Drafting and Lateral Drafting configurations, the drafter experiences lower fatigue and larger swimming efficiency than in Free-Swimming.
- Behind Drafting is more advantageous than Lateral Drafting.
- The benefits of Lateral Drafting over Free-Swimming are likely to depend on the drafter’'s ability to take advantage of the wave region created by the leader.

## Introduction

Drafting in swimming is a competitive tactic in which an athlete (drafter) swims in the wave of another athlete (leader), taking advantage of the water turbulence generated by the leader to experience a lower drag in gliding through the water environment (Bentley et al., 2007).

In indoor swimming competitions, the effect of drafting is not relevant, because the athletes swim in different lanes and the presence of anti-turbulence lane ropes contributes to minimizing the wave turbulence substantially (Yuan et al., 2019). The advantage of the draft emerges instead in the open water swimming and triathlon competitions.

The effectiveness of drafting has been investigated by the direct measurement of passive drag (i.e., by comparing the force required to tow a drafter in streamlined position through the water at constant speed in drafting and non-drafting conditions) as well as by measuring metabolic and kinematic parameters of the drafter, in different experiment designs, varying the distance between the drafter and the leader, their active or passive role, their swimming speed, and other variables (Bassett et al., 1991; Chatard et al., 1998; Chollet et al., 2000; Millet et al., 2000; Chatard and Wilson, 2003; Delextrat et al., 2003, 2005; Bentley et al., 2007; Silva et al., 2008; Janssen et al., 2009; Arfaoui and Polidori, 2014; Salihu et al., 2016; Yuan et al., 2019).

Compelling evidence from experiments performed at triathlon race pace (average velocity of 1.24 m s^−1^) indicates that the most advantageous drafting position to minimize drag is when the drafter’s hand is almost touching the leader’s feet (Behind Drafting, BD; Millet et al., 2000; Chatard and Wilson, 2003; Janssen et al., 2009).

In BD, Janssen et al. (2009) investigated leader’s dynamics using a pilot tube system win a counter-current swimming flume. With respect to the case with no leader (Free Swimming, FS), the drafter experienced a 20% reduction in passive drag with a “passive” leader in front—i.e., a leader who was holding onto a rope while maintaining a streamlined position—and a 10% reduction when the leader was actively swimming (“active” leader). However, on the negative side, it was suggested that BD with an active leader could induce a visual disadvantage for the drafter, due to the bubbles created by the leader’s beat kicks (Millet et al., 2000).

Swimming sideways and behind the leader is an alternative position for the drafter. To make the most of the advantage, the optimal position is when the drafter’s head is aligned with the leader’s hip (Lateral Drafting, LD; Chatard and Wilson, 2003; Janssen et al., 2009). When LD was performed with a passive leader, the decrease of passive drag in the drafter (in comparison to FS) was about one-third than detected in BD. However, when LD was performed with an active leader, in the drafter an unexpected increase of passive drag was reported (Janssen et al., 2009).

Besides the reduction of drag, decreased metabolic load (oxygen uptake, lactate level, and heart rate), lower perceived exertion, increased stroke length and swimming speed, and decreased stroke rate were reported in the drafter (Bassett et al., 1991; Chatard et al., 1998; Chollet et al., 2000; Chatard and Wilson, 2003; Delextrat et al., 2005; Janssen et al., 2009). Changes in metabolic load and kinematic parameters, as well as perceived exertion, may be a consequence of muscle fatigue, defined as “*a reduction in force output that occurs during sustained voluntary activity*” (Bigland-Ritchie et al., 1983). However, to our knowledge, muscle fatigue in the drafter was never investigated using surface electromyography (EMG), which allows a more direct (related to force output) and selective (related to single muscles or muscle groups) assessment of muscle fatigue in real-time (Cifrek et al., 2009).

Electromyography is a widely established method to assess muscle fatigue in terms of the frequency components of the EMG signal. During fatigue, because of the accumulation of catabolites such as inorganic phosphate and phosphocreatine, the acidity of the interstitial fluid increases causes a change in the shape of the action potential and a decrease of the muscle-fiber conduction velocity (Brody et al., 1991). These physiological changes shift the EMG power spectrum to low frequencies so that the time evolution of the Mean Frequency of the EMG signal [MNF (Hz)] is a reliable parameter to estimate muscle fatigue (Puce et al., 2021a). The Root Mean Square of the EMG signal [RMS (μV)] is another parameter used in the analysis of muscle activity (Del Vecchio et al., 2017). RMS is directly related to the muscle force output (Sale, 1987). It has been used by several authors as an ancillary measure of muscle fatigue (Stirn et al., 2011; Puce et al., 2021b).

The main aim of this study was to investigate and compare the muscle fatigue of the drafter during the 200 m front crawl test, performed in the following three experimental swimming configurations: BD, LD, and FS. These muscles were selected based on previously published studies assessing their main function in swimming propulsion (Pink et al., 1991; Rouard et al., 1997; Ikuta et al., 2012; Figueiredo et al., 2013). The second aim is to assess the muscle activity and swimming efficiency associated with muscle fatigue by means of RMS and kinematic parameters.

The outcome measures of this study were: (1) time slope of MNF, to evaluate muscle fatigue; (2) time slope of RMS, to evaluate muscle activity; and (3) Stroke Index (SI), to evaluate swimming efficiency.

Based on the above-reported literature, we hypothesize lower muscle fatigue, larger swimming efficiency, and lower muscle activity in BD, in comparison with both LD and FS.

## Materials and Methods

### Participants

Eighteen swimmers competing at either interregional or national level took part in the research study, 11 men and one woman as both drafters and leaders, three women as drafters only, and three men as leaders only. Each drafter was behind the same leader for all the drafting configurations. The subjects were both middle- and long-distance swimmers with experience in swimming pool and open water competitions. The mean and SD of age, body mass, and height for both drafters and leaders are reported in Table 1. The study was carried out in accordance with the code of ethics of the World Medical Association (Declaration of Helsinki 2014) for experiments involving humans. Written informed consent was obtained from all participants prior to participation in the study. The project was approved by the local ethics committee (University of Genova, Italy. No. 2020/21).

**Table 1:** Personal characteristics of the participants. Values for age, mass, and height are defined as average and ±SD.

Participants	Total number of subjects in both sexes	Sex female	Sex male	Age (year)	Mass (kg)	Height (m)
Leader	15	1	14	20.4 ± 4.3	72.1 ± 7.2	1.8 ± 0.2
Drafter	15	4	11	20.0 ± 3.1	69.4 ± 8.3	1.7 ± 0.2

### Experimental Design

**Figure 1:**
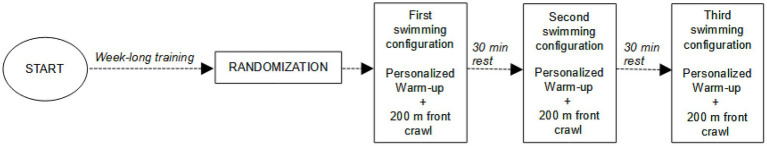
Sketch of experimental design. The timeline of the study is the following: (1) week-long training to learn to follow the metronome pace and keep the correct position in the drafting configurations; (2) randomization of the participants in the different swimming configurations; (3) three 200 m front crawl trials, each trial in different swimming configurations. Each trial was preceded by a warm-up with a 30-min recovery between successive trials.

The swimming test consisted of three 200 m front crawl trials at a controlled submaximal pace in three different swimming configurations: BD, LD, and FS. The testing set was preceded by a warm-up with a 30-min recovery between successive trials. The order of the swimming configurations was counterbalanced across participants using a computer-generated randomization order (Figure 1).

During the swimming test, muscle fatigue (MNF), muscle activity (RMS), and swimming efficiency (SI) were acquired by EMG and video analysis from flexor carpi radialis (FCR), triceps brachii (TB), latissimus dorsi (Lat.D), and rectus femoris (RF) muscles.

### Swimming Test

Measurements were performed in a 25 m indoor swimming pool. Drafter performed the three 200 m front crawl trials, each trial in different swimming configurations: BD, LD, and FS. The leader performed only two of the 200 m front crawl trials, those in BD and LD. Drafters swam at a similar pace in all three swimming configurations. The pace was set to a swimmer’s personal best over 1,500 m distance. Leaders adapted their speed to the drafters’ needs. During the test, participants (both drafters and leaders) wore a metronome (Finis Tempo Trainer, Livermore, CA, United States) under the swimming cap at the height of the temple to help keep the predefined swimming pace. The time interval among metronome’s audio feedbacks was adjusted to coincide with the time required to swim from side to side of the pool length (25 m), according to the set pace. To ensure constant swimming speed throughout the trials, participants carried out a week-long training to learn to follow the metronome pace and keep the correct position in the drafting configurations. In the extant scholarly literature, van Houwelingen et al. (2017) have evaluated arm-leg coordination in breaststroke technique, while Yamakawa et al. (2017) have studied the underwater dolphin kick phase with or without synchronization of kick frequency with the beat of a metronome. However, to the knowledge of the authors, there is no research that uses the metronome to establish the rhythm in swimming.

Furthermore, video footage of the athletes swimming across the 5–20 m segment of the pool was recorded during the trials and then checked for inconsistencies in the swimming speed (see section related to Kinematic data). If any velocity variation was found, the corresponding trial was removed from the data.

**Figure 2:**
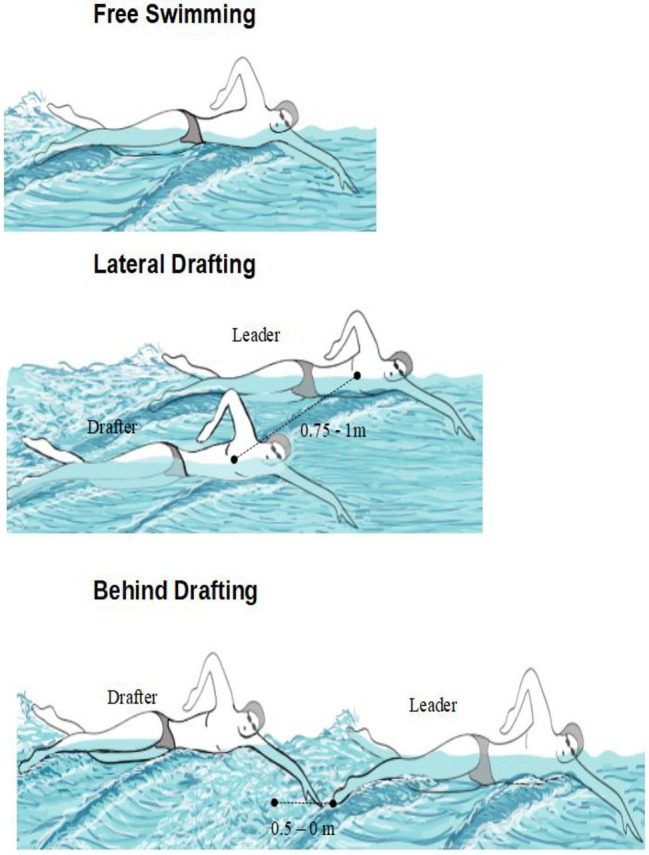
Sketch of the reciprocal position of drafter and leader in the three configurations of the experiment.

In BD, the drafter swam behind a leader between a distance of 0 m (the fingertips of the drafter almost touched the toes of the leader) and 0.50 m (0.50 m between toes of the leader and fingertips of the drafter). To avoid collisions in turns the leader was required to slightly deviate their trajectory in the push from the wall. In LD, the drafter swam with the head at the level of the drafter’s hip, with a lateral distance of 0.75–1 m between bodies’ midlines (Figure 2).

Due to the measuring equipment attached to the body (EMG electrodes and adhesive markers), the underwater turn was allowed, but the dive start was not.

### EMG Data

Electromyography signals from FCR, TB, Lat.D, and RF of the dominant side were measured through bipolar surface electrodes using waterproof wireless EMG equipment (Cometa srl, Milan, Italy) operating at 2,000 Hz, according to the SENIAM guidelines (Hermens et al., 2000). To avoid alterations induced by underwater recording, a water-resistant adhesive tape (Fixomull transparent, BSN medical, Hamburg, Germany) was applied over the electrodes. The EMG intervals were filtered with a band-pass Butterworth filter of fourth order in the range of 20–500 Hz. Data analysis was performed using the open-source software Python distributed by Anaconda Inc. For each muscle (FCR, TB, Lat.D, and RF), the activation interval corresponding to the under-water propulsive phase of each stroke was identified where the envelope of the rectified signal around the maximum amplitude exceeded 20% of the maximum amplitude itself, following the same criterion of Stirn et al. (2011). Once the starting and ending times of the activation interval of each muscle in each stroke (*t*_in_ and *t*_fin_) were identified, evaluation of MNF and RMS values were carried out on each activation interval. Performing this operation gave a plot of MNF and RMS values vs. timwe. In order to estimate the time evolution of MNF and RMS, a linear fitting of the data set was performed, and the slope was extracted.

Following the same procedure of Ikuta et al. (2012), EMG signals exhibiting noise (approximately ≥3SD), as well as homologous signals for all participants, were rejected for data analysis.

Mean frequency was calculated as the momentum of order 1 of the power spectrum:


(1) $$MNF=\frac{\int_{f_{c1}}^{f_{c2}}f\cdot PSD\left(f\right)df}{\int_{f_{c1}}^{f_{c2}}PSD\left(f\right)df}$$

where *f*_c1_ = 20 Hz and *f*_c2_ = 500 Hz are the cutoff frequencies of the high-pass and low-pass filters applied to the spectra and PSD(*f*) is the power spectral density [PSD(*f*)].

The RMS amplitude was calculated as:


(2) $$RMS=\frac{1}{\left(t_{\mathrm{fin}}-t_{\mathrm{in}}\right)}\overset{t_{\mathrm{fin}}}{\underset{t_{\mathrm{in}}}{\int }}{\left(f\left(t\right)\right)}^{2}dt$$

Time variations of MNF and RMS were measured from the slope of the linear regressions of these parameters vs. time, where time data were the initial times (*t*_in_) of the activation intervals of each stroke. The slopes were finally normalized to the value of the regression line at the initial time of the first analyzed activation interval for each EMG trace. Average values of normalized slopes were calculated by weighting each value with the respective inverse variance $\frac{1}{\sigma_{i}^{2}}$ obtained from the linear regression (*N* corresponding to number of participants):


(3) $$x_{\mathrm{average}}=\frac{\sum_{i=1}^{N}\frac{x_{i}}{\sigma_{i}^{2}}}{\sum_{i=1}^{N}\frac{1}{\sigma_{i}^{2}}}$$

The error bars on these averages were calculated as:


(4) $$\sigma_{x}=\sqrt{\frac{1}{\sum_{i=1}^{N}\frac{1}{\sigma_{i}^{2}}}}$$

### Kinematic Data

Kinematic data were obtained by analyzing video recordings (Kinovea 0.8.25), acquired on sagittal plane using two cameras (model GoPro Hero 8, GoPro, San Mateo, CA, United States), one above the water surface and the other underneath. The cameras were fixed to a pushcart, which was moved at the same speed as the participants. Information on the position of body and limbs of the drafter was obtained by applying adhesive markers on the joints of the lower and upper limbs and synchronizing the biomechanical analysis with the EMG signal. Specifically, triggering of video recording and EMG signal was done by tapping a spare EMG probe at the start.

Stroke index was defined as the product of speed and stroke length (SL). SL was calculated by dividing the speed by stroke rate (SR). The time required to complete five stroke cycles was used to calculate SR. All these metrics were determined in the free-swimming segment (i.e., from the 5th to the 20th meter of the pool) of each length of the 200 m using video recordings of the athletes swimming across the aforementioned segment. The latter was delimited by markers underwater and on the surface.

### Statistical Analysis

One-way ANOVA test was used to evaluate the significance of differences of muscle fatigue, muscle activity, and swimming efficiency between different swimming configurations. Statistical significance of the normalized slopes of MNF and RMS for each muscle, averaged over the participants, was evaluated by a single sample *t*-test, testing the difference between the value and zero.

*p* value thresholds for increasingly larger statistical significance were set to *p* < 0.05, *p* < 0.001, and *p* < 0.0001. Normal distribution of data (both RMS and MNF) was checked by the Shapiro-Wilks test.

## Results

Due to excessive noise caused by the EMG probe with the surface of the water during the stroke, seven activation signals were rejected for ANOVA tests with homogeneous groups (four signals rejected for RF, two for FCR, and one for TB).

**Figure 3:**
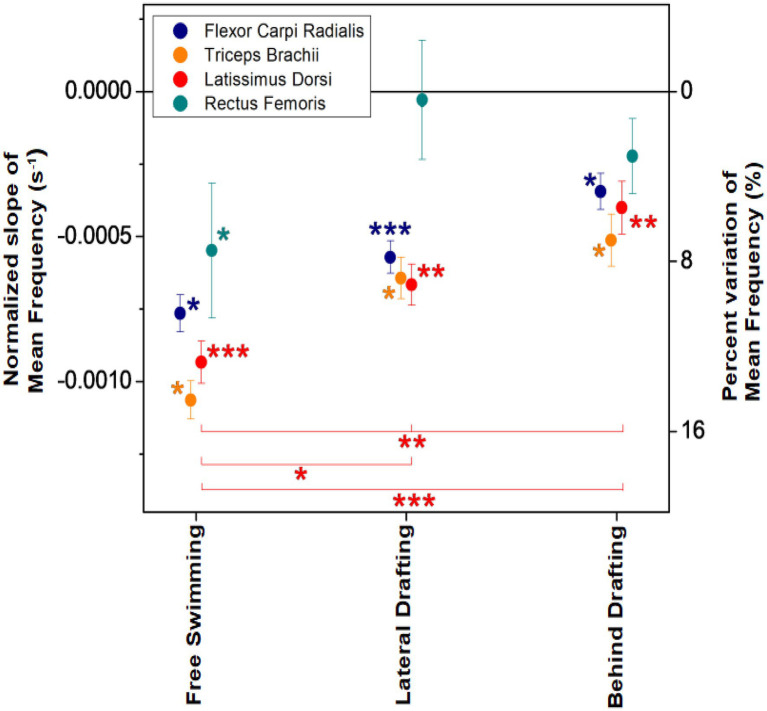
Normalized slopes of Mean Frequency (MNF) for each of the four muscles and for each of the three swimming configurations, averaged over the participants. In the right-hand axis, the corresponding percent variations in the 200 m test are indicated. Statistical significance of average slopes and of difference between average slopes among two or three configurations is indicated by asterisks (^*^ for *p* < 0.05, ^**^ for *p* < 0.001, and ^***^ for *p* < 0.0001).

In Figure 3, we present the normalized slopes of MNF for each muscle and for each of the three configurations, averaged over the participants. It can be seen that negative slopes were found in all cases, and statistical significance was achieved in all cases except to RF data in the two drafting configurations (BD and LD). The corresponding variations of MNF in the 200 m tests were 5.1 ± 1.7, 6.6 ± 4.1, and 11.1 ± 2.7% in BD, LD, and FS, respectively. The differences of MNF slopes between swimming configurations were statistically significant only for Lat.D, and specifically statistical significance was found for the difference between the three configurations FS-LD-BD (*p* = 0.000432, *F* = 10.3481) and between FS-LD (*p* = 0.000432, *F* = 10.3481) and FS-BD (*p* = 0.0000827, *F* = 25.5401) pairs of configurations.

**Figure 4:**
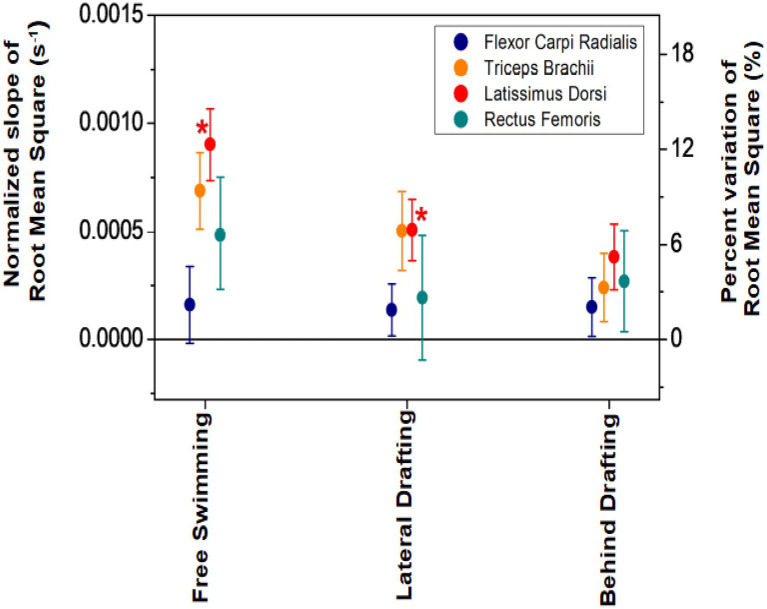
Normalized slopes of Root Mean Square (RMS) for each of the four muscles and for each of the three swimming configurations, averaged over the participants. In the right-hand axis, the corresponding percent variations in the 200 m test are indicated. Statistical significance of average slopes is indicated by asterisks (^*^ for *p* < 0.05).

In Figure 4, we present the normalized slopes of RMS for each muscle and for each of the three configurations, averaged over the participants. Positive values were found for all the muscles and swimming configurations, and corresponding variations of RMS in the 200 m tests were 3.4 ± 1.2, 4.7 ± 2.7, and 7.8 ± 4.6 in BD, LD, and FS, respectively. Statistical significance was found only for the slopes of Lat.D in the FS and LD configurations. The average slopes among configurations were not statistically significant.

**Figure 5:**
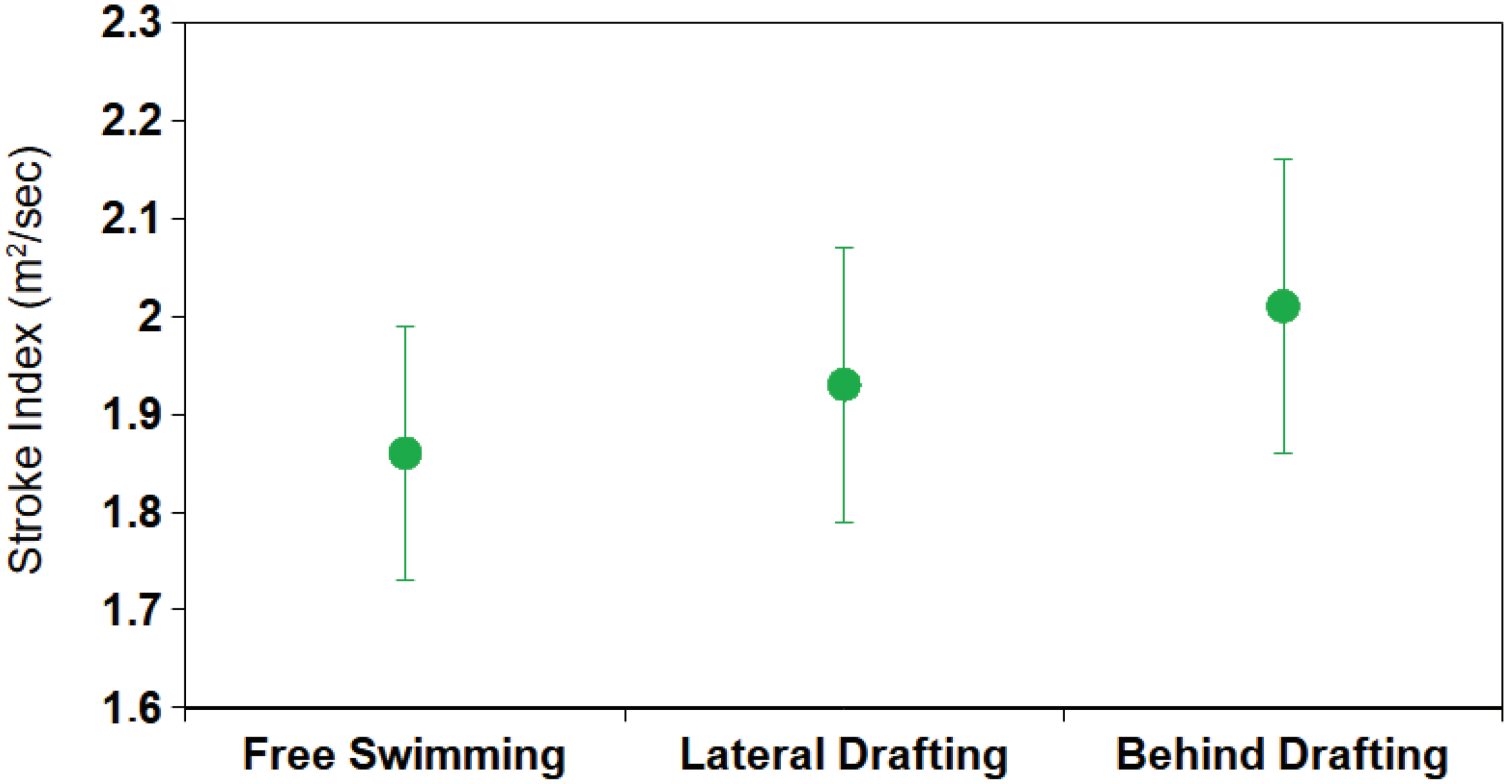
Stroke Index (SI) for each of the three swimming configurations, averaged determined in the free-swimming segment (i.e., from the 10th to the 20th m of the pool).

Stroke index values showed neither appreciable nor systematic time evolution throughout the 200 m tests, for this reason, the mean values are representative of each test. In Figure 5, the mean SI for each of the three swimming configurations, averaged over the participants, are shown. The largest mean SI was measured in the BD configuration (2.01 m^2^/s), while the smallest was measured in the FS configuration (1.86 m^2^/s). SD is around 0.14 m^2^/s and the differences among configurations were not statistically significant.

## Discussion

In this study, we evaluated muscle fatigue and associated changes in swimming efficiency and muscle activity in three configurations of swimming (BD, LD, and FS). The results of this study support our hypothesis that in BD the drafter experiences lower fatigue and has larger swimming efficiency than in LD and in FS. In addition, as expected, also LD resulted to be a more advantageous configuration than FS.

### Muscle Fatigue and Muscle Activity

During the swimming test (three 200 m front crawl trials), muscle fatigue manifested in decreased MNF and increased amplitude of EMG activity (RMS).

Generally speaking, the increase of RMS mirrors the additional recruitment of motor units and is linearly related to the production of mechanical force (Sale, 1987). However, as far as our results are concerned, no appreciable changes in swimming efficiency and speed were detected in the swimming test also due to the specific study design. Therefore, we can assume that the additional recruitment of motor units did not act to increase the production of mechanical force, but instead to compensate for muscle fatigue (Edwards and Lippold, 1956). Indeed, in submaximal motor tasks, the increase of RMS is considered an indirect measure of muscle fatigue (Petrofsky, 1979; González-Izal et al., 2012). According to this largely accepted view (Puce et al., 2021b), we assume that our athletes recruited additional motor units to maintain pre-fatigue force output in order to keep velocity and swimming efficiency constant. Therefore, lower increase of RMS and lower decrease of MNF indicate lower fatigue in the drafting configurations in comparison to FS.

Comparing muscle fatigue in different muscles, a similar trend was found in each swimming configuration. The largest muscle fatigue (i.e., the largest negative MNF slope) was observed for Lat.D and TB. According to the studies focused on EMG amplitude in front crawl muscles (Stirn et al., 2011; Martens et al., 2015), Lat.D and TB are those more involved in producing the propulsive force. Indeed, Lat.D is known as the “*swimmers muscle*” due to its major role in the successful completion of each of the swim styles (Laudner and Williams, 2013) and, together with TB, it is considered the key muscle in maintaining swimming speed in fatigued conditions (Ikuta et al., 2012). On the contrary, RF exhibited lower fatigue than the other muscles. This can be explained by the fact that lower limbs contribute only by ~15% to the front crawl propulsion (Toussaint et al., 2006; Seifert and Chollet, 2008; Stirn et al., 2011), and their contribution is further reduced for long-distance specialists who use their lower limbs not so much as a propulsive thrust, but rather for buoyancy. Finally, regarding FCR, an intermediate level of fatigue was observed. In front crawl propulsion, the contribution of this muscle is limited to stabilizing the wrist with the forearm in the early pull-through phase (Caty et al., 2006).

### Hydrodynamic Interactions

Janssen et al. (2009) found an increase in passive drag in LD, as compared to FS. The authors attributed these results to the large waves formed by the active leader, which may create areas that are hydrodynamically unprofitable for the drafter. These findings seem to be in contrast with our results showing advantages provided by LD. However, this discrepancy could depend on the drafter’s ability to take advantage of the region of waves created by the leader. Swimmers with year-long experience in open water competitions probably have the ability, when they are drafters in LD, to swim in a wave-riding configuration, i.e., with the upper body in the wave trough and the lower body in the wave crest (Figure 1). As observed by Yuan et al. (2019) in ducklings following their mother and in dolphins chasing boats, this positioning is likely to exert a forward pull on the swimmer riding the wave—a phenomenon generated by the pressure difference between wave crest (high pressure) and wave trough (low pressure), resulting in a pressure gradient along the swimmer’s body and thus creating propulsion (Yuan et al., 2019).

### Practical Implications

In open water competitions, a drafter can profit from both: a leader of equal swimming capability and a leader of superior level. In the first case, the wave created by the leader will enable the drafter to follow even when swimming at a pace slightly slower than what a non-drafting configuration would require. This allows for the alleviation of the effort during the competition and for better energy management. In the second case, drafters can use the wave to elevate their pace beyond their normal ability, which can enable them to keep up with swimmers of higher capacity.

In training, the concept is identical. However, when the leader has the same level of ability as the drafter, the coach must be aware that the drafter will fatigue less and thus may not be in the physiological condition the training aims at. Given the importance of the drafter’s ability to swim in wave-riding arrangement, LD should be addressed in specific training sessions with progressive difficulty. It is advisable to begin training in an environment free of currents and waves and with reference points such as the swimming pool. Only after that the transition to open water should be made.

### Limitation of This Study

As mentioned in the “Materials and Methods” and in the “Results,” EMG signals (more specifically their power spectrum) can be altered by the impact that is generated when the body part the EMG probe is attached to breaks the water surface. Furthermore, water can alter the conductivity between skin and electrode by strongly variating the amplitude and frequency of the EMG signal (Rainoldi et al., 2004).

To account for this circumstance, water-resistant adhesive tape has been applied to the electrodes (see the section “EMG data”). However, some activation signals showed excessive noise and were rejected for ANOVA tests with homogeneous groups (four signals rejected for RF, two for FCR, and one for TB). Only for Lat.D, all the acquired signals were included in the analysis because none of them had excessive noise. We believe this factor explains the lack of statistical significance of some data, despite clear trends being observed (see Figure 3). For example, statistical significance of ANOVA tests, applied to different drafting configurations with a threshold level of 0.05, was reached neither by datasets of MNF for muscles other than Lat.D, nor by datasets of RMS for any muscle. The results of the ANOVA tests depend not only on the mean and SD values but also on the number of samples, and the latter probably made the difference between the statistical significance of the Lat.D. compared to other muscles.

To address this limitation, further technological advances are needed to minimize the amount of noise, an EMG probe records during water-based trials.

Another limitation of this study, to be considered in view of generalizing our results to open water contexts, is that factors like the presence of currents, weather conditions, group dynamics (with more than two athletes), and absence of turns and underwater phases were completely neglected. Testing the different swimming configurations under the presence of these factors should provide further valuable insight into their respective advantages and drawbacks. We recommend that future studies investigating this matter should use the fatigue assessment method presented in this work, as we believe it enables to capture an athlete’s fatigue level in a comprehensive way (MNF, RMS, and kinematics) and as there is no reason to believe that the mentioned factors could interfere with its functionality.

A further limitation of the present investigation is that, given its exploratory nature, we did not consider other muscles that could be important in crawl, such as pectoralis major.

## Conclusion

The present study indicates that drafting is an effective tactic for the drafter in terms of muscle fatigue and swimming efficiency. BD was found to be the best swimming configuration.

## Data Availability Statement

The original contributions presented in the study are included in the article/supplementary material, further inquiries can be directed to the corresponding author.

## Ethics Statement

The studies involving human participants were reviewed and approved by IRB of University of Genoa, Genoa, Italy. The patients/participants provided their written informed consent to participate in this study.

## Author Contributions

LP conceived and designed the experiment, and collected data. NLB analyzed data. KC, LMa, MB, EF, MF, FC, and CT drafted and critically revised the paper. All authors contributed to the article and approved the submitted version.

## Conflict of Interest

The authors declare that the research was conducted in the absence of any commercial or financial relationships that could be construed as a potential conflict of interest.

## Publisher’s Note

All claims expressed in this article are solely those of the authors and do not necessarily represent those of their affiliated organizations, or those of the publisher, the editors and the reviewers. Any product that may be evaluated in this article, or claim that may be made by its manufacturer, is not guaranteed or endorsed by the publisher.
